# Histone Acetyltransferase and Deacetylase Inhibitors—New Aspects and Developments

**DOI:** 10.3390/ijms242316985

**Published:** 2023-11-30

**Authors:** Hany S. Ibrahim, Wolfgang Sippl

**Affiliations:** Department of Medicinal Chemistry, Institute of Pharmacy, Martin Luther University of Halle Wittenberg, 06120 Halle (Saale), Germany

Epigenetic processes modulate gene transcription and genomic stability, ensuring proper cell development and differentiation. Epigenetic processes are controlled, among other things, by post-translational modifications on histones, with acetylation/acylation being a highly abundant example of these modifications. Acetylation/acylation patterns on histones as well as numerous non-histone proteins are regulated by the synergy between a group of epigenetic enzymes, histone acetyltransferase enzymes (HATs), which introduce the modification, and a group that removes the modification, named histone deacetylases (HDACs). Histone deacetylases, in particular, have come into focus for drug discovery in recent years, and the first five inhibitors have been approved for cancer therapy. Although these compounds are non-selective HDAC inhibitors, current research is focused on developing selective inhibitors of the 18 HDAC subtypes. In particular, the recently explored subtypes, such as HDAC10, HDAC11, or the Sirtuins, represent new targets for putative drugs. In addition, more and more acyl groups have recently been found to be removed from individual HDAC subtypes, including crotonyl, palmitoyl, or other fatty acid derivatives. The availability of crystal structures for most of the HDACs also enables the application of in silico and structure-based approaches to drug discovery. In the case of histone acetyltransferases, much fewer inhibitor discovery studies have been reported so far.

A completely new approach is the inhibition of physiological processes via protein degradation. This can be achieved with so-called proteolysis targeting chimera (PROTAC) or molecular glues. PROTACs are heterobifunctional molecules that couple a target ligand with a ligand for an E3 ligase so that the enzyme is polyubiquitinated and degraded by the proteasome. For HDACs, the PROTAC field is still in its infancy.

The aim of this Special Issue was to highlight current efforts and new results in medicinal chemistry and biological characterization of histone deacetylase and acetyltransferase inhibitors as well as degraders. Several studies were included to discover the applications of such biological processes and the results of modulating the acylation or deacylation processes by inhibition or degradation. In addition, several studies have been published in which combinations of HDAC/HAT inhibitors with other drugs have been evaluated for their therapeutic efficacy.

As mentioned above, the biological effect of HDACs extends to various aspects, as shown in the included review article (contribution 1) describing the expression and regulation of individual subtypes of HDACs on the different stages of T cell development. With regard to givinostat, a pan-HDAC inhibitor, a study was conducted (contribution 2) to investigate the hypothesis that HDAC inhibition by givinostat contributes to the reduction in inflammation after neonatal hypoxia-ischemia (HI). Another study focusing on combination therapy was reported by de Macedo et al. (contribution 3). The results showed that the combination of the HDAC inhibitor Scriptaid with the inhibition of RNA synthesis in somatic cell nuclear transfer (SCNT) in porcine embryos increases the efficiency of gene editing and cell reprogramming.

The search for potential and selective inhibitors for the different isoforms of HDACs is also a challenge, as so far, all FDA-approved HDAC inhibitors are effective but not selective, which can lead to severe side effects. To investigate the structural aspects for the development of selective HDAC8 inhibitors, the role of Met274 as a selectivity factor for specific HDAC8 inhibitors was investigated by mutational studies and biophysical measurements (contribution 4). To allow rapid measurement of HDAC inhibition in in vitro assays, Zessin et al. (contribution 5) presented a novel continuous assay based on developed peptide substrates with extraordinary kinetic constants compared to usually applied substrates.

Three articles in the Special Issue deal with the effects of inhibitors that are selective for HDAC1, 2, and 3 and how these selective HDAC inhibitors behave in combination with other drugs. First, Chen et al. (contribution 6) illustrated the effect of a combination of chidamide, as a class I HDAC inhibitor, with both VEGFR and PD1-directed compounds and the effect of this combination on the tumor microenvironment. This study showed that the addition of chidamide to the VEGFR inhibitor regorafenib and anti-PD-1 antibody combination could induce a durable tumor-specific response by attenuating immune suppression in the tumor microenvironment in murine colon carcinoma. Second, Susetyo et al. (contribution 7) discussed the effect of the HDAC3 inhibitor RGFP966 on the cerebellar morphological defects in perinatal hypothyroid mice. The inhibitor was shown to induce the hyperacetylation of histones and promote transcription at thyroid hormone-responsive gene loci, potentially acting as a novel therapeutic treatment for cerebellar abnormalities caused by hypothyroidism. Another study focusing on the selectivity topic by Wang et al. (contribution 8) found that BG45, as a selective HDAC3 inhibitor, possessed and extended therapeutic effects towards Alzheimer disease by reducing inflammation and controlling the CREB/BDNF/NF-kB pathway. 

Another reported zinc-binding moiety resulting in class II selective compounds, the 1,2,4 trifluoromethyoxadiazole (TFMO) moiety, was used in the study by Bollmann et al. (contribution 9). They developed a TMFO-based class IIa inhibitor (YAK540) and analyzed the combined effect of both YAK 540 and bortezomib, a proteasome inhibitor, to induce apoptosis in leukemic cells.

A different mode of action was tested in the study by Darwish et al. (contribution 10) who pursued the development of proteolysis targeting chimera (PROTAC) for HDAC8 and the investigation of their activities against neuroblastoma. It has been shown that the developed PROTAC strongly degrades the HDAC8 protein in neuroblastoma cells. Since it is known that inhibition of HDAC8 in neuroblastoma cells induces signs of neuronal differentiation, such as neurite outgrowth, the combination of the most promising PROTAC with the known neuronal differentiation inducer retinoic acid (ATRA) was investigated. It was shown that the combination significantly enhances the differentiation phenotype compared to the use of one substance alone.

In addition, several papers in this issue focused on the effects of modulating epigenetic targets in the context of acetylation and deacetylation processes. Lisi et al. (contribution 11) reported on the effect of acetylation of histone (H3K9ac) using the endogenous scFv-58F on gene expression and compared this effect with two established HAT inhibitors. Yoon et al. (contribution 12) illustrated the effect of overexpression of choline acetyltransferase by transplantation on the improvement of cognitive functions with simultaneous ovarian removal in rats to avoid the effect of the estrogen receptor. In addition to acetylation, crotonylation of non-histone lysine residues affected the browning of white fat in rats and was therefore discovered by Liu et al. (contribution 13) as a promising means to control obesity. Finally, a review article highlighting the role of EP300 as an integral regulatory protein in fibrosis through the acetylation of histone proteins was published in this issue (contribution 14).

Overall, the research presented in this Special Issue demonstrates how different experimental methods can contribute to the development of selective HDAC inhibitors, the elucidation of their effects in combination with other therapeutics, and the detailed biological characterization of the cellular effects of these treatments.

